# Meigs Syndrome and Elevated CA-125: Case Report and Literature Review of an Unusual Presentation Mimicking Ovarian Cancer

**DOI:** 10.3390/medicina59091684

**Published:** 2023-09-19

**Authors:** Irene Iavarone, Michela Padovano, Francesca Pasanisi, Luigi Della Corte, Elvira La Mantia, Carlo Ronsini

**Affiliations:** 1 Department of Woman, Child and General and Specialized Surgery, University of Campania “Luigi Vanvitelli”, 80138 Naples, Italy; ireneiavarone2@gmail.com (I.I.); padovanomichela29@gmail.com (M.P.); pasanisi.francesca@gmail.com (F.P.); carlo.ronsini@unicampania.it (C.R.); 2 Department of Neuroscience, Reproductive Sciences, and Dentistry, University of Naples Federico II, 80131 Naples, Italy; 3 Pathology Unit, Department of Mental and Physical Health and Preventive Medicine, University of Campania “Luigi Vanvitelli”, 80138 Naples, Italy; elvira.lamantia@unicampania.it

**Keywords:** Meigs syndrome, ascites, hydrothorax, CA-125, ovarian neoplasms

## Abstract

*Background and Objectives*: Meigs syndrome is represented by a benign adnexal tumor, ascites, and hydrothorax. Even though the ovarian mass is often characterized by a fibroma-like origin, cancer antigen-125 (CA-125) serum levels could be elevated as in the development of ovarian cancer. Here, we present the case of a patient with Meigs syndrome and increased CA-125. *Materials and Methods*: We performed systematic research for articles including similar cases in PubMed, EMBASE, and Scopus in February 2023, adopting the string of idioms: “Meigs syndrome AND Cancer antigen 125”, and following the Preferred Reporting Items for Systematic Reviews and Meta-Analyses (PRISMA) statement. *Results*: Eligible records were 25. Hydrothorax was right-sided in 10 cases over 25; left-sided in two patients over 25. Concerning ascites, two patients showed more than 6 L of ascitic fluid, whereas three patients had 6 L or less. CA-125 elevation ranged from 149 IU/mL to 3803 IU/mL. Adnexal mass histotypes were: struma ovarii (12 cases), thecomas (two cases), fibrothecomas (five cases), fibromas (five cases), and one sclerosing stromal tumor (SST). *Conclusions*: In postmenopausal women with elevated CA-125 serum levels and an adnexal mass suspicious for malignancy at ultrasound (US), ascites and pleural effusion, surgery, and histopathological examination are necessary. MS is a diagnostic option, with an excellent prognosis after exeresis of the mass.

## 1. Introduction

Meigs Syndrome (MS) is a rare presentation defined as the triad of a benign ovarian tumor with ascites and pleural effusion. It is a sporadic syndrome with a low incidence rate. Regarding the ovarian tumors, only 1% present as an MS [1,2,3]. The MS involves a solid benign adnexal formation—fibroma, thecoma, granulosa cell tumor—associated with hydrothorax and ascites. In particular, ascites and pleural effusion spontaneously dissolve after mass exeresis [4]. CA-125 (cancer antigen-125), also known as MUC16 or Mucine 16, is a human glycoprotein employed for the diagnosis and follow-up of different cancer histotypes: first, epithelial ovarian cancer (EOC) [5]. Commonly, it is slightly raised in MS, although in the scientific literature, a value above 1000 IU/mL is rare [6,7,8]. The present work is a case report of MS with elevated CA-125 levels. The aim of the work is also to conduct a systematic review of the scientific literature to evaluate similar cases and raise awareness of a rare clinical entity.

### Case Presentation

**Figure 1:**
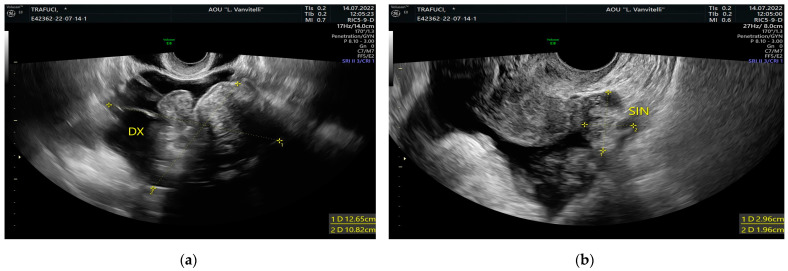
Transvaginal ultrasound image of adnexa: (**a**) right (e.g., *DX*) adnexal mass; (**b**) normal left (e.g., *SIN*) ovary.

**Figure 2:**
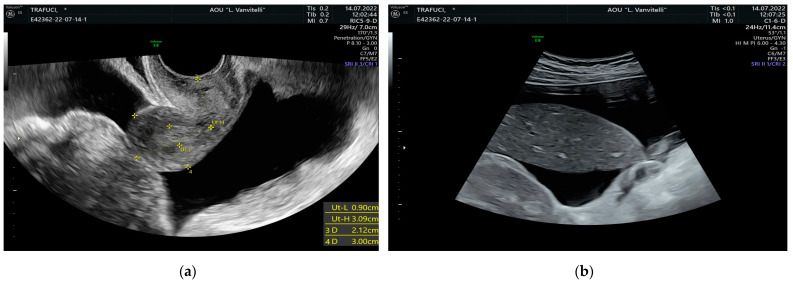
Transvaginal ultrasound image of massive ascites: (**a**) in the pouch of Douglas; (**b**) in the perihepatic space.

A 61-year-old Italian woman, administered with levothyroxine 75 mcg for hypothyroidism only, denied other pathologies and previous pregnancies and had been complaining about abdominal pain and swelling for 6 months. The patient underwent a complete abdominal computed tomography (CT) scan of the abdomen and pelvis in another institution without and with contrast, which showed a “voluminous pelvic mass” (maximum diameters 136 × 71 mm) in the median area. The mass was inhomogeneous, with weakly vascularized solid components and hypodense components with a cystic aspect. The origin of that form appeared to be adnexal at first hypothesis, with conspicuous ascitic effusion in the supramesocolic and inframesocolic peritoneal spaces, with a slight thickening of omental fat in the ventral area. In particular, this is suspicious for carcinosis in postmenopausal women. No significant lymph node swellings were reported. The patient was admitted to our department with increased abdominal volume due to ascites and pelvic mass. These were noticed through transvaginal ultrasound (US), which showed an extensive adnexal solid mass measuring 126 × 87 mm, located in the pelvis and apparently of right adnexal origin (Figure 1a). The aspect of the left ovary seemed regular at the transvaginal US (Figure 1b). Massive ascites was noticed both in the pouch of Douglas and in the perihepatic space (Figure 2a,b). Blood tests were performed, and serum CA-125 levels were increased to 1644.5 IU/mL (normal range: 5–35 IU/mL), whereas serum levels of other cancer antigens like CA 19-9, CA 15-3, carcinoembryonic antigen (CEA), alpha-fetoprotein (AFP), and Inhibin B were into the normal range. The patient started to be dyspneic; oxygen saturation was less than 92% in spontaneous breathing. A chest examination revealed an absence of breath sounds and dullness to percussion upon the right lung, suggesting effusion. Shortly after, a right thoracic drainage was positioned due to pleural effusion, leading to 1200 mL of citrine yellow fluid aspiration. The clinicians opted for a diagnostic laparoscopy followed by eventual cytoreduction, based on carcinomatosis suspicion, considering the faster recovery time and minor length of hospital stay. Laparoscopy with an open Hasson trans-umbilical technique was performed. Before introducing the optical system, 1800 mL of ascitic fluid was drained, and albumin was administered to the patient shortly after. In the abdomen, no carcinomatosis was seen. Omental fat, stomach, and bowel appeared free from nodules. The pelvis was not completely visible due to the presence of the pelvic mass. It was solid, with smooth and regular margins, measuring about 20 cm. It rose from the right ovary. Unilateral right salpingo-oophorectomy was performed, and the surgical specimen was sent for histopathological examination. The latter revealed a benign ovarian fibroma. The aspect of the uterus and contralateral ovary was regular (Figure 1b) and the primary plan of a hysterectomy was abandoned [9]. No further surgical maneuver was performed based on the patient’s consent. Estimated blood loss was close to 0 mL. The woman was readmitted to the Gynecology Department and discharged home in good clinical condition and spontaneous breathing, with no evidence of ascites. Final histology reported “Ovary measuring 13 × 8 × 4 cm and tube 5 cm long with two para-tubal cysts. Smooth outer surface. In section, compact, fasciculated area with several edematous aspects; laminar cystic formation with 5 cm maximum diameter, filled with clear serous fluid. Those are the morphologic findings for ovarian fibroid-like sex-cord tumor”. The cytology of pleural effusion was not performed. CA-125 levels were 657 IU/mL on the first day after surgery. Blood test repetition one month after the reported negative values; hence, the patient was considered in remission. Moreover, we systematically searched the literature to find a similar presentation of that syndrome.

## 2. Materials and Methods

The methods for this study were specified a priori based on the recommendations in the Preferred Reporting Items for Systematic Reviews and Meta-Analyses (PRISMA) statement [10].

### 2.1. Search Method

We performed systematic research for records about cases of MS with elevated CA-125 serum levels in PubMed, EMBASE, and Scopus in February 2023. We made no restriction on the country nor the year of publication, and we only considered studies published entirely in English. We adopted the following string of idioms in each database to identify studies which were fitting to the topic of our review: “MS AND Cancer antigen 125”.

### 2.2. Study Selection

The study selection was made independently by M.P. and I.I. In case of discrepancy, C.R. decided on inclusion or exclusion. The inclusion criteria were based on: (1) studies describing cases of MS and elevated CA-125 serum levels; (2) peer-reviewed articles published originally. We excluded: non-original studies, pre-clinical trials, animal trials, abstract-only publications, and articles in a language other than English. If possible, the authors of studies that were published as conference abstracts were contacted via e-mail and asked to provide their data.

## 3. Results

**Figure 3:**
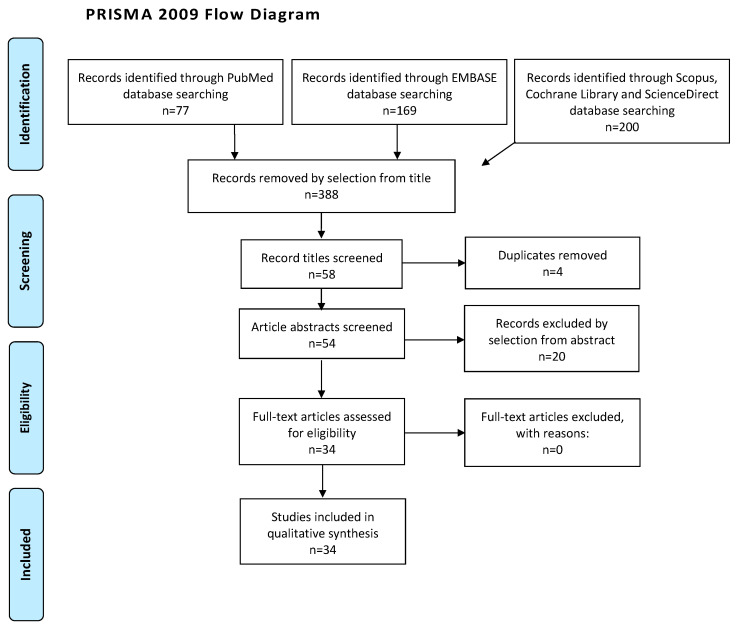
Preferred Reporting Items for Systematic Reviews and Meta-Analyses (PRISMA) flow diagram.

We mentioned the studies selected and all reasons for exclusion in the Preferred Reporting Items for Systematic Reviews and Meta-Analyses (PRISMA) flowchart (Figure 3). We assessed all included studies concerning potential conflicts of interest.

### Studies’ Characteristics

After the database search, 446 articles matched the search criteria. After removing records with no full-text available, duplicates, abstracts unfitting to the topic of our review, and wrong study designs (e.g., reviews), 38 were suitable for eligibility. Of those, 34 matched the inclusion criteria and were included in the systematic review (Figure 3). The countries where the studies were conducted, the studies’ design, the enrollment year range, and the number of participants are summarized in Table 1. Overall, the publication years ranged from 1995 to 2022. In total, 36 patients with MS and elevation of CA-125 were included in the systematic review. Table 1 shows that patients’ ages ranged from 13 to 78 years old. Of the 36 selected patients, 28 presented data about hydrothorax and all records revealed information regarding ascites. The former was right-sided in 16 patients over 36, left-sided in three cases over 36, and bilateral in five cases, whereas the remaining articles did not mention hydrothorax location. Regarding ascites, two patients presented with an amount of ascitic fluid greater than 6 L, whereas eight patients had 6 L or less. In six cases, ascites amount was described as massive, whereas the remaining 20 cases did not mention ascites amount. Elevation of CA-125 ranged from 42.3 IU/mL to 3969 IU/mL. Adnexal mass histotypes were 12 struma ovarii, 2 thecomas, 6 fibrothecomas, 14 fibromas, 1 sclerosing stromal tumor (SST), and 1 granulosa cell tumor (GCT).

## 4. Discussion

The entity of an ovarian mass, ascites, and hydrothorax was recognized for the first time in 1887 by Demons and in 1937 by Meigs [43]. For that reason, it is also known as Demons-MS [43]. The most common histological types in the scientific literature are cellular fibroma, fibro-thecoma, fibroma, thecoma, and granulosa cell tumors [6]. Over the years, pseudo-MS has also emerged [31]. It shows the same clinical features of MS, but the adnexal mass is not fibroma-like. Still, it could be mucinous cystadenoma, teratoma, ovarian metastasis (mainly from colorectal cancer), leiomyoma, or struma ovarii [31,44]. Although various cases of MS with elevated CA-125 serum levels have been described over the years, only 1% of ovarian fibromas or fibrosarcomas have presented with ascites and pleural effusion [31,44,45,46]. For that reason, MS is considered rare in that specific context [31,44,45,46]. The serum levels of CA-125 ranged from 149 IU/mL to 3803 IU/mL, and only two patients presented with CA-125 levels higher than 2000 IU/mL [11,37]. CA-125 is a glycan produced by the uterus, cervix, fallopian tubes, and cells that line the respiratory tract and abdominal organs. However, the precise mechanism related to CA-125 elevation in the context of MS is unclear [1]. One possible explanation may be the irritation with subsequent inflammation of the serosal surface—pleura and peritoneum—provoked by free fluid in the mediastinal and abdominal cavities [1]. Damage or inflammation of those tissues causes the elevation of CA-125, the same as the development of ovarian tumors [1]. The CA-125 cancer marker is generally higher in women with malignant adnexal masses [47]. However, it can also be elevated in benign conditions like endometriosis, pelvic inflammatory disease, uterine fibroids, the menstrual cycle, early pregnancy, liver disorders, or pancreatitis [47,48,49]. Moreover, serum CA-125 levels can also increase in the case of pericardial, pleural, and peritoneal irritation or inflammation [1,50]. In parallel, the elevation of CA-125 levels was detected in 29% of women with non-gynecological malignancies and non-oncological afflictions [51]. There are different hypotheses explaining the formation of ascites. The leading theory is that the transudation of fluid through the tumor surface exceeds the proportion for the peritoneal reabsorption process [6]. Another theory supports the congestion of peritoneal lymphatic vessels and veins, mainly caused by the mass and, secondly, by vasoactive mediators released by the mass itself [6] Regarding hydrothorax constitution, the most probable mechanism underlies the passage of ascitic fluid through the diaphragm and lymph nodes into the chest, and then into pleural virtual space, leading to a pleural effusion [2]. Vascular Endothelial Growth Factor (VEGF) seems to be associated with pleural and peritoneal fluid formation because it increases vascular permeability [52]. Moreover, we hypothesize the possibility of using other biomarkers for the recognition of an ovarian mass. Today, MS is mainly identified through the triad of a benign ovarian tumor, ascites, and pleural effusion. This means that MS can be identified mostly through signs and the symptoms. Based on the presented cases, there is no consent on the entity of the ascites and hydrothorax. In fact, ascites range from 9 L to 2.2 L, whereas hydrothorax distributes in both lungs or in one lung, with no apparent correlation to any other variable, as represented in Table 1. Unfortunately, the description of symptoms is poor in the reported cases. The patient who was referred to our department did not complain about pelvic pain, or other gynecological symptoms. When the entity of the hydrothorax and ascites is massive, the patients may show thoracic and abdominal swelling. In addition, regarding CA-125 serum levels, as shown in Table 1, we did not find any apparent relation with other key elements of MS. Those data demonstrate the difficulty in diagnosing MS. In particular, our patient was referred to the hospital showing a CT-scan performed in another institution that did not specialize in gynecological disorders.

MS presentation can be classified into: Classic, Non-Classic and Demons-Meigs’, and Pseudo-Meigs [53]. The Classic form includes the presence of a benign fibroma or fibroma-like mass, such as granulosa cell tumor, thecoma, Brenner tumor, ascites, pleural effusion, and the complete resolution of the ascites and pleural effusion after the exeresis of the mass [53]. The Non-Classic MS classification is used when ascites and pleural effusion coexist with a benign ovarian tumor or with a Fallopian tube or broad ligament tumor [53]. Other authors sustain that the present definition should be equated to the Classic one [54]. In particular, that theory was initially supported by Albert Demons [43]. Our systematic review also included patients with the Non-Classic form of MS, excluding women with Fallopian tube tumor and broad ligament tumor. Pseudo-Meigs Syndrome involves ascites and pleural effusion depending on other pelvic or abdominal tumors and is not to be included in the Demons-Meigs Syndrome criteria [53]. That condition is subcategorized according to its benign or malignant connotation [53]. The former regards all the benign pelvic or abdominal masses situated outside the ovaries, the Fallopian tubes, and the broad ligaments, whereas the latter regards primary or metastatic neoplasms of the pelvis or abdomen [53]. In those contexts, any peritoneal or pleural spread should be detected through negative cytology or also through negative malignant cells in biopsy samples [55]. Moreover, ascites and pleural effusion must be resolved after the removal of the mass [44,55,56]. In addition, so-called Atypical or Incomplete MS shows either ascites or pleural effusion coexisting with the mass [57]. It must be classified into Meigs’, Demons-Meigs’, or Pseudo-Meigs’ Syndrome according to the characteristics of the tumor [57,58,59].

Further perspectives can focus upon the use of liquid biopsy or even the composition of intestinal microbiota, in order to identify the phenotype of an ovarian mass, especially if that has an endometriotic nature [60,61]. In parallel, Meigs Syndrome with elevated CA-125 levels mimics ovarian cancer, even though it remains a benign condition. Therefore, it would be appropriate to improve the diagnostic options through the use of additional non-invasive biomarkers before surgery. Obviously, liquid biopsy and miRNA identification can be performed upon various substances. In the case of the triad of MS, that technique might also be applied on ascites and pleural effusion. Hypothetically, the same miRNAs should be detected both in pleural and peritoneal fluid, because we expect them to be related to CA-125 elevation. Otherwise, miRNA expression may differ based on the nature of the ovarian mass.

In addition, it would be interesting to examine the colonized microbial composition of pleural and peritoneal fluids, in order to identify a key profile. To detect pleural effusion in women, a feasible option is the pleural fluid analysis [62,63,64]. In particular, the examination of the different fluid components and properties offers reliable information upon the mechanism underlying the fluid accumulation [53]. The most common distinction of the fluid’s nature is based on transudate and exudate [53]. However, Bayesian analysis provides a more specific ratio of the exudative effusion [65]. The rarity of MS makes it extremely challenging to establish the main features of the pleural fluid; hence, it becomes important to associate that presentation with the great entity of dyspnea and abdominal swelling, which are present in 32% of MS-affected women [53]. In fact, 95.4% of patients with malignant Pseudo-Meigs Syndrome underwent at least one thoracentesis [53]. In general, the exudate is more common in MS compared to transudate [2,62,63]. The distinction transudate and exudate can be performed through the puncture Rivalta test and Light’s criteria [53]. In the Rivalta test, a tube is filled with distilled water and acetic acid. Afterwards, one drop of the effusion is added. If the drop dissolves, the test is negative and indicates a transudate; however, if the drop precipitates, the test is positive and indicates an exudate [66]. Light’s criteria combine three dichotomous experiments comparing the number of proteins and LDH between the serum and the fluid [67]. In general, the proportion of proteins is the most affordable criterion to discriminate between the exudative or transudative nature of MS fluid [53]. Based on the evidence of the scientific literature, MS is mostly associated with an exudate [53]. Unfortunately, we did not perform any of those tests on pleural effusion. The main theory supporting the formation of pleural effusion regards the accumulation of the ascitic fluid throughout the diaphragmatic pores [68,69]. However, the accumulation of peritoneal fluid is far less investigated. Probably, it is linked to the serum levels of inflammatory cytokines, which augment the vessels’ leakage [70]. The increased vascular permeability may also depend on the Vascular Endothelial Growth Factor (VEGF), Fibroblast Growth Factor (FGF), and Interleukin-6 (IL-6) release [70]. There is evidence demonstrating that the serum levels of those molecules decrease after the removal of the mass, and their reduction is associated with the resolution of ascites and hydrothorax [52]. VEGF is usually produced by tumor cells, but it can be secreted by extra-tumor sources like ovarian metastases [70,71]. Otherwise, ascites may be the result of an excessive interstitial edema [72]. The interstitial fluid accumulates into the abdominal cavity due to an imbalance between the arterial blood supply to an extensive tumor and the venous drainage [72]. Moreover, the lymphatic vessels—covered by a single-layer cuboidal epithelium—are not able to hold the fluid and it escapes into the peritoneal cavity [72].

One of the main limitations of our study is linked to the rarity of MS. Cases reported in the scientific literature are presented heterogeneously, especially regarding the entity of ascites and pleural effusion. Moreover, the cytology of the fluids is not always described.

On the other hand, our work could raise awareness of an unusual presentation of a systemic disease involving multiple comorbidities, and thus requiring a wider choice of treatment. 

In the case of MS, it is important to evaluate the patient’s age, in order to choose the best surgical and staging options. Our patient was 61 years old; however, other cases reported in the scientific literature show more heterogeneous findings [73,74].

## 5. Conclusions

In postmenopausal women with elevated CA-125 serum levels suspicious for a malignant adnexal mass at US visualization, signs like ascites and pleural effusion, surgery, and histopathological examination are required. These are necessary for the correct diagnosis and treatment of ovarian tumors. Indeed, MS could be a diagnostic option, showing an excellent prognosis after mass exeresis.

## Figures and Tables

**Table 1:** Summary of different cases of MS with elevated CA-125 levels.

Author, Year of Publication	Age	Country	Period of Enrollment	Hydrothorax	Ascites	CA-125 (IU/mL)	Histotype
Siddiqui 1995 [11]	57	USA	1995	Minimal right-sided	Straw-colored 7.6 L	3184	Struma ovarii
Timmerman 1995 [12]	71	Belgium	N/A	Massive right-sided, minimal left-sided	1 L	484.5	Fibroma
	73	Belgium	N/A	N/A	0.5 L	42.3	Fibroma
Bethune 1996 [13]	46	Australia	1996	Marked bilateral	Moderate straw-colored 6 L	1230.9	Struma ovarii
Abad 1998 [14]	50	Spain	1998	Right-sided	Present	1476.8	SST
Fujii 2002 [15]	N/A	Japan	N/A	Present	Present	Raised	Fibroma
Huh 2002 [16]	52	USA	2002	Moderate right-sided	Present	149	Struma ovarii
Renaud 2002 [17]	57	Canada	N/A	N/A	Straw-colored 9 L	1750	Thecoma
Bokhari 2003 [18]	65	USA	2003	Right-sided	Massive	161	Struma ovarii
Vieira 2003 [6]	51	Brazil	2003	Bilateral	Present	577	Fibroma
Korkolis 2004 [19]	65	Greece	2004	Moderate bilateral	6 L	402	Struma ovarii
Loizzi 2005 [20]	52	Italy	2005	Present	Present	319.2	Fibrothecoma
Jung 2006 [21]	62	Korea	2006	Present	Present	Raised	Fibrothecoma
Peyron 2006 [22]	51	France	2006	Right-sided	Present	412	Fibrothecoma
Obeidat 2007 [23]	52	Jordan	2007	2 L bilateral	Massive 2.2 L	1289	Struma ovarii
Mitrou 2008 [24]	65	United Kingdom	2008	Left-sided	Massive	406.3	Thecoma
Benjapibal 2009 [25]	56	Thailand	N/A	1.5 L right-sided	2.5 L	1064	Fibroma
Jiang 2010 [26]	72	China	2010	Massive right-sided	Present	607.4	Struma ovarii
Liou 2011 [27]	78	Taiwan	2011	N/A	Present	164	Struma ovarii
Monteiro 2012 [28]	50	Portugal	2012	Present	Present	600	Fibrothecoma
Mostaghel 2012 [29]	13	Iran	2012	Moderate right-sided	Voluminous	453	Fibroma
Yilmaz 2012 [30]	40	Turkey	N/A	Sera-hemorragic	0.5 L	823	Fibroma
Cha 2014 [31]	N/A	South Korea	2014	Right-sided	Absent	Raised	Fibroma
Yazdani 2014 [32]	50	Iran	N/A	Left-sided	Present	600	Fibrothecoma
Ghani 2015 [33]	50	Bangladesh	2015	Right-sided	Clear 2 L right-sided hydronephrosisand	355.1	Fibroma
Jin 2015 [34]	73	China	2014	Right-sided	Present	1780	Fibroma
Laan 2016 [35]	49	The Netherlands	N/A	Present	Present	Raised	Fibrothecoma
Park 2016 [36]	72	Korea	N/A	N/A	Massive	327	Fibroma
Sofoudis 2016 [37]	55	Greece	2016	Bilateral basal lung atelectasis with a small left pleural effusion	Massive	3803	Struma ovarii
Oluwasola 2017 [38]	62	Nigeria	2017	Massive right-sided	Small	1621	Struma ovarii
Yadav 2017 [39]	55	India	2016	N/A	Present	258	Struma ovarii
Navarro-Esteva 2020 [40]	59	Spain	N/A	Minimal right-sided	Absent	1000	Fibroma
Khanduja 2021 [41]	50	India	N/A	Right-sided	Absent	534	Fibroma
	48	Spain	N/A	Pale-yellow right-sided	Present	526	GCT
Liu 2022 [4]	N/A	China	2021	Present	Present	Raised	Struma ovarii
Ushida 2022 [42]		Japan	2022	N/A	Massive	3969	Fibroma

CA-125: cancer antigen-125; L: liters; SST: sclerosing stromal tumor; GCT: granulosa cell tumor.

## Data Availability

Data supporting the Results can be found in References.
